# Expression of nutrient transporters in placentas affected by gestational diabetes: role of leptin

**DOI:** 10.3389/fendo.2023.1172831

**Published:** 2023-07-11

**Authors:** Pilar Guadix, Isabel Corrales, Teresa Vilariño-García, Carmen Rodríguez-Chacón, Flora Sánchez-Jiménez, Carlos Jiménez-Cortegana, José L. Dueñas, Víctor Sánchez-Margalet, Antonio Pérez-Pérez

**Affiliations:** ^1^ Obstetrics and Gynecology Service, Virgen Macarena University Hospital, School of Medicine, University of Seville, Seville, Spain; ^2^ Clinical Biochemistry Service, Virgen del Rocio University Hospital, School of Medicine, University of Seville, Seville, Spain; ^3^ Clinical Biochemistry Service, Virgen Macarena University Hospital and Department of Medical Biochemistry and Molecular Biology and Immunology, School of Medicine, University of Seville, Seville, Spain

**Keywords:** gestational diabetes mellitus, placental transport, leptin, glucose, amino acids, lipids, fetal macrosomia, nutrient transporters

## Abstract

Gestational diabetes mellitus (GDM) is the most frequent pathophysiological state of pregnancy, which in many cases produces fetuses with macrosomia, requiring increased nutrient transport in the placenta. Recent studies by our group have demonstrated that leptin is a key hormone in placental physiology, and its expression is increased in placentas affected by GDM. However, the effect of leptin on placental nutrient transport, such as transport of glucose, amino acids, and lipids, is not fully understood. Thus, we aimed to review literature on the leptin effect involved in placental nutrient transport as well as activated leptin signaling pathways involved in the expression of placental transporters, which may contribute to an increase in placental nutrient transport in human pregnancies complicated by GDM. Leptin appears to be a relevant key hormone that regulates placental transport, and this regulation is altered in pathophysiological conditions such as gestational diabetes. Adaptations in the placental capacity to transport glucose, amino acids, and lipids may underlie both under- or overgrowth of the fetus when maternal nutrient and hormone levels are altered due to changes in maternal nutrition or metabolic disease. Implementing new strategies to modulate placental transport may improve maternal health and prove effective in normalizing fetal growth in cases of intrauterine growth restriction and fetal overgrowth. However, further studies are needed to confirm this hypothesis.

## Graphical Abstract

Nutrient transport in normal and altered conditions.

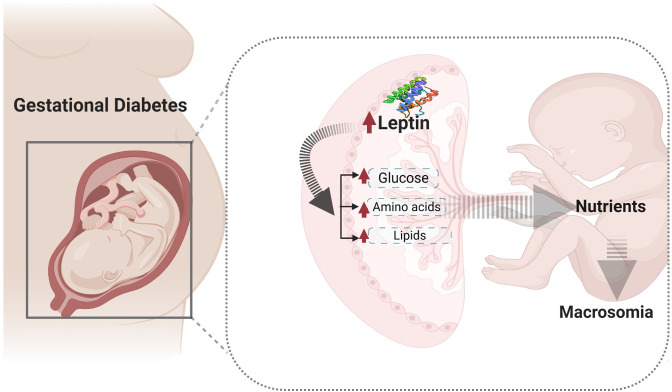

## 1 Introduction

Gestational diabetes mellitus (GDM) is a condition characterized by glucose intolerance that occurs during pregnancy (1). It is one of the most common complications, affecting 3–8% of all pregnancies (1, 2). The prevalence of GDM has increased in recent decades, reaching rates of ≥20% in some parts of the world, due to factors such as increased maternal age and obesity (3). In Spain, the estimated prevalence of GDM ranges from 3 to 5% of pregnancies, depending on factors such as ethnicity, age, fetal sex, and diagnostic criteria. It is considered the leading cause of fetal macrosomia, which is associated with an increased risk of perinatal mortality and neonatal morbidity (4, 5).

The placenta from GDM subjects is often susceptible to altered metabolism, which could change the expression of placental nutrient transporter systems (6). In fact, this change may disrupt fetal growth and expose offspring to long-term consequences of GDM. Consequently, the offspring of women affected by GDM are more likely to suffer from long-term conditions such as obesity, cardiovascular disease, metabolic syndrome, and type 2 diabetes mellitus later in life (7). Therefore, it has been hypothesized that fetal development is more closely linked to altered placental transport resulting from GDM-associated conditions rather than maternal circulation.

An array of maternal circulating factors has been shown to alter placental macronutrient metabolism in pregnancies complicated by GDM (8). For example, a wide range of hormones and cytokines such as insulin, leptin, insulin-like growth factors (IGFs), and molecular signaling pathways, including the mammalian target of rapamycin (mTOR), phosphatidylinositol 3-kinase (PI3K), and mitogen-activated protein kinases (MAPK), have been reported to be activated in the maternal-facing microvillous (MVM) of human placentas affected by GDM (6, 9, 10). Based on these facts, the activity of placental nutrient transport might be modulated by the maternal environment and placental metabolism (11). Therefore, since the human placenta is responsible for the production of adipokines (12), leptin has been implicated in various related pathologies of pregnancy such as GDM, obesity, fetal growth abnormalities, and metabolic dysfunction (6, 13, 14).

Maternal-fetal macronutrient transport is primarily mediated by a family of placental transporters. For example, glucose transport, in particular, is mediated by glucose transporters (GLUTs) (15), while amino acid transport is mediated by several membrane transport proteins, including the sodium-dependent system A transporter, also called the serotonin N-acetyltransferase (SNAT) family and the sodium-independent system L transporter, known as the large neutral amino acid transporters (LATs) family (16, 17). On the other hand, lipid transport is mediated by the free fatty acid transporter (FATB), fatty acid-binding proteins (FABPs), and the activity of lipases in the MVM of syncytiotrophoblast (18).

During pregnancy, placental leptin levels increase in pregnancies complicated by GDM (19). This suggests a physiological role for leptin in the placental uptake of nutrients. However, the mechanism by which leptin may act as a modulator of placental transporter expression in GDM pregnancies is not well understood. Recently, leptin and insulin signaling pathways have been demonstrated in placentas from GDM pregnancies (20), suggesting a role for leptin in the expression of placental transporters in GDM. However, data and evidence on this topic are limited, and therefore it is not fully understood.

Previous studies have shown that leptin increases the glucose uptake rate in cultured trophoblasts by increasing GLUT1 expression in the human placenta BeWo cell line (21, 22). Additionally, leptin increases the expression of Aquaporin-9 (AQP9), an aquaglyceroporin membrane channel, in normal trophoblasts (23), providing a substrate for gluconeogenesis. Increased expression of AQP9 has been found in placental trophoblasts from patients with GDM (24). Leptin has also been implicated in placental amino acid uptake. In this regard, several studies have reported that leptin stimulates the system A transporter in villous fragments via PI3K and the JAK (Janus tyrosine kinase)-STAT (signal transducer and activator of transcription) (JAK/STAT) pathway in obese pregnant women (25, 26). These data suggest a role of leptin in regulating amino acid metabolism in the human placenta and probably in placentas affected by GDM linked to maternal obesity (7). Concerning placental fatty acid transport, little is known about the leptin effect in GDM. However, in normal pregnancy, it has been shown that leptin increased fatty acid translocase, such as FAT/CD36, expressions in the syncytiotrophoblast of human placentas in primary cell cultures (27).

Taking all these facts together, we aimed to review the reported placental transport changes during GDM and to elucidate the possible role/impact of leptin on placental transporter expressions altered by GDM.

## 2 Gestational diabetes mellitus

GDM is one of the most common complications of pregnancy (28, 29) and affects 6–25% of pregnant women (depending on diagnostic criteria) (30). Although the GDM phenotype is highly heterogeneous (28), half of its prevalence can be explained by being overweight and obesity (3). GDM is associated with an increased risk of stillbirth and neonatal death, as well as multiple serious morbidities for both the mother and baby (28). The main pathophysiological complications of GDM are due to fetal macrosomia, which is characterized by a larger placenta weight and size to support the increased needs of the macrosomic fetus. The alteration of placental function may be a reason for abnormal fetal growth (29) observed in this pregnancy pathology (30). However, its pathophysiology is not fully clarified yet. In this sense, women with GDM have increased plasma leptin levels (31). Additionally, insulin levels are also increased in GDM, and hyperinsulinemia may mediate an increase in leptin synthesis in the placenta (32). In fact, the role of leptin in the growth and metabolism of the placenta has been demonstrated (9), where the trophic action of leptin may mediate an increase in the size of placentas affected by GDM (6, 33), as both the expression of leptin and its receptor were increased (34).

### 2.1 Diagnostic criteria of GDM

Unfortunately, there is no scientific consensus on the best way to diagnose GDM due to discrepancies in the definition criteria. Expert professional worldwide organizations acknowledge several acceptable options (35, 36), each with its own advantages and disadvantages. According to the American Diabetic Association and the National Institute for Health and Care Excellence, the diagnosis of GDM is defined as glucose intolerance diagnosed in the second or third trimester of pregnancy. Both criteria consider a fasting plasma glucose level ≥ 5.6 mmol/L or a 2-h plasma glucose level ≥ 7.8 mmol/L (7).

In Spain, a screening approach is used [preferred by the National Diabetes Data Group (NDDG) (37)] for gestational diabetes at week 24 of gestation, employing the O’Sullivan test (oral glucose tolerance test (OGTT) with 50 g of glucose). This includes an initial non-fasting 1-h glucose challenge test (35), which is logistically simpler for patients and can easily be performed as part of a scheduled prenatal visit. Most women do not require further screening. However, approximately 20% of patients fail this screening [values ≥ 140 mg/dL (≥ 7.77 mmol/L)], and a diagnostic test is then carried out using an OGTT of 100 g, with assessment of glycemia at baseline and at 3 h (38). Two or more increased values are diagnostic for GDM.

In the first trimester of pregnancy, and if there are risk factors (family history of diabetes, previous child weighing more than 4,500 g, age > 35 years, or history of gestational diabetes in a previous pregnancy), an O’Sullivan test is required. If the O’Sullivan test is pathological, the OGTT with 100 g is performed. If the result is normal, the OGTT with 100 g would be performed again in the second trimester.

### 2.2 Risk factors of GDM

GDM is a complex pathophysiological state of pregnancy in which both genetic and environmental factors are involved. Maternal obesity is considered the main risk factor for developing GDM (7). Indeed, obese women have an increased risk of GDM compared to women of normal weight (28). Therefore, both obesity and GDM lead to fetal overgrowth, which is associated with an increased capacity for maternal nutrient supply across the placenta to the fetus (11). Nevertheless, many other factors could contribute to a high risk of GDM, such as a family history of any form of diabetes, advanced maternal age, race (particularly non-white), or having previously given birth to large babies. Moreover, pathologies related to insulin resistance and leptin resistance, such as polycystic ovarian syndrome (PCOS) and pre-eclampsia, may also lead to GDM (39, 40).

The consequences of GDM during pregnancy can have significant impacts on both the mother and the newborn later in life. Castillo et al. have recently described the short-term and long-term complications of GDM in their work (7). Short-term complications may include maternal pre-eclampsia, fetal macrosomia, shoulder dystocia, and higher body fat. Additionally, complications during delivery, such as prolonged labor, caesarean birth, surgical complications, hemorrhage, infection, and extended hospital stays, may also occur in some cases. Moreover, the long-term complications of GDM can increase the risk of both the mother and her child developing type 2 diabetes mellitus, cardiovascular disease, metabolic syndrome, and obesity (7) later in life.

## 3 Leptin

Leptin is the satiety hormone secreted by adipose tissue and plays a crucial role in regulating energy balance (41). In fact, circulating leptin levels reflect adipose tissue size and also change with the nutritional state. Furthermore, leptin is considered a pleiotropic hormone that regulates not only body weight but also many other functions, including the immune system and the systemic inflammatory response, as well as the normal physiology of the reproductive system (9, 42). In this sense, leptin is involved in the ovulatory cycle, where it acts to maintain the energy balance linked to this process (43). Therefore, leptin can act as a metabolic switch connecting the nutritional status of the body to high energy-consuming processes. This is especially important in pregnancy, where leptin not only modulates satiety and energy homeostasis in the mother (41), but is also produced by the placenta. This is why, during pregnancy, high leptin secretion levels have been detected in both maternal and fetal circulations, which rise until childbirth (44, 45). The serum leptin concentration is two- to three-fold higher in pregnant women compared to nonpregnant counterparts and peaks at approximately 28–32 weeks of gestation. Immediately after delivery, levels rapidly decrease back to pre-gestational levels (46). This significant increase in maternal circulating leptin during pregnancy is caused by increased leptin production from adipose tissue in connection with weight gain and fat deposition in the mother from the late second trimester onwards, as well as by placental leptin production in the first trimester, which constitutes approximately 15% of the total maternal serum leptin concentration (47). In this sense, leptin modulates the dialogue between fetal and maternal metabolism (48). The leptin expression increases in the human (and non-human primate) placenta as pregnancy proceeds (49).

### 3.1 Leptin in normal pregnancy

Leptin is not only an adipokine hormone secreted by adipose tissue but also an autocrine trophic factor produced by trophoblast cells to regulate the growth and metabolism of the placenta (50–55). Leptin also plays an essential role in reproduction by regulating gonadotropin-releasing hormone secretion (56). Moreover, overexpression of leptin in animal models has been reported to cause early puberty (57). In humans, maternal leptin levels have been shown to be higher in the early stages of pregnancy (45). This data was later confirmed in 2004 by Nuamah et al., where the authors demonstrated an increase in maternal leptin levels during induced delivery, along with an increase in placental leptin mRNA production (58). The various reproductive functions of leptin include the regulatory control of different processes, such as placental growth, macronutrient transport, placental angiogenesis, trophoblast mitogenesis, and immunomodulation (6, 14), all crucial events for fetal development and adequate placental function.

### 3.2 Leptin in pathologic pregnancy

During pregnancy, the majority of plasma leptin is secreted by the placenta. The increased level of placental leptin has been linked to several metabolic conditions during pregnancy, such as maternal obesity and gestational diabetes mellitus (GDM) (6). Leptin and its receptors are key regulatory factors implicated in various pregnancy complications. Its role has been further studied in pregnant women with obesity, GDM, and pre-eclampsia. A summary of the more relevant implications of leptin in pregnancy complications is provided in **Table 1**.

**Table 1:** Summary of the most common pregnancy complications and the role of leptin in their pathophysiology.

Pregnancy disease-related	Role of leptin.	Expressions	References
Maternal obesity	Increased leptin levels in obese pregnant women are significantly correlated with fetal overgrowth, leading to macrosomia.	↑	(59)
Pre-eclampsia	Leptin is a predictive marker of pre-eclampsia in obese pregnancies. Increased placental leptin levels in all trimesters of pregnancy represent risk factors for pre-eclampsia. Higher maternal leptin levels increase oxidative stress in high-altitude (HA) residents with pre-eclampsia. Lower serum leptin levels decrease placental production when pre-eclampsia is associated with HIV infection. Lower leptin levels in newborn babies from pre-eclamptic pregnancies are significantly correlated with obesity and oxidative stress.	↓,↑	(60–64)
Intrauterine growth restriction (IUGR)	Higher maternal leptin levels may contribute to fetal distress. Increased leptin concentration in prepubertal males born with IUGR is emerging as an early marker of metabolic syndrome. Maternal serum leptin and endothelin-1 (a highly potent vasoconstrictor released in conditions of hypoxia) was found to be increased and positively correlated with the degree of fetal growth restriction in women with pre-eclampsia. Lower leptin levels are associated with intrauterine growth restriction (IUGR) in discordant dichorionic twins. The evidence strongly correlates lower leptin concentrations with pregnancies complicated by IUGR, leading to fetal malnutrition.	↑,↓	(65–67) (68)
Recurrent miscarriage	Leptin gene polymorphisms at different levels may increase the risk of recurrent spontaneous abortion	↑	(69, 70)
Polycystic ovary syndrome (PCOS)	Hyperleptinemia is significantly associated with PCOS and may lead to an increased free leptin index in young women. Increased leptin levels, along with other biochemical parameters, contribute to increased oxidative stress in adolescent girls with PCOS. The leptin signaling pathway plays a crucial role in the production of male hormones in women with PCOS. Leptin and insulin resistance mediate lower expression of aromatase in granulosa cells from women with PCOS.	↑	(39, 40, 71–73)

↑, increase in expression levels; ↓, decrease in expression levels.

Pregnancies complicated by GDM are under the influence of many regulatory factors such as growth hormones, insulin resistance, and leptin, which may impact placental transport and fetal growth (7). Maternal obesity and the differences in diagnosis criteria may also impact the accuracy of the available data related to the involvement of leptin in the regulation of the placental transporter mechanism altered by GDM (7). Currently, several studies consider hyperleptinemia to be a good parameter in predicting GDM in early pregnancy (6, 19, 74). In addition, another group have shown that leptin levels are higher in women with early onset of GDM during pregnancy compared with standard onset and overweight women (75). Moreover, the authors of the same paper mentioned that these women had an inflammatory profile. Therefore, leptin is likely involved in the inflammatory response during pregnancy complicated by GDM.

An array of metabolic and endocrine alterations, such as hyperinsulinemia, hyperleptinemia, and oxidative stress, have been linked to pregnancy complicated by GDM. In this sense, Shang et al. reported that the increase of leptin levels in GDM was significantly correlated with markers of oxidative stress such as malondialdehyde, 8-isoprostane, and xanthine oxidase (76). Furthermore, the increase of leptin levels in GDM is more likely linked to body mass index (BMI) and is probably expected to alter fetal growth, leading to high fat body in GDM offspring. In this context, recent studies have demonstrated that higher leptin levels are associated with insulin-resistance in newborns from mothers with GDM (77, 78). Additionally, Powe et al. have demonstrated that an adjustment of leptin levels in early pregnancy enhances the insulin secretory response in GDM women (79). Such a result may indicate the possible beneficial role of leptin in GDM therapy.

GDM is the most common metabolic disorder that may alter placental nutrients transport. GDM is mainly characterized by insulin-resistance and leptin-resistance (10). Thus, higher concentrations of placental leptin mRNA and protein have been shown in placentas affected by GDM compared to healthy controls (13), and placental leptin production is increased in GDM. In this context, leptin is a regulatory factor that can modulate placenta functions in an autocrine or paracrine manner, involved in many processes such as proliferation and protein synthesis (51, 53, 80–82) and probably placental transporter activity or expression. Thus, the role or impact of leptin on placental transporters of macronutrients such as glucose, amino acids, and lipids in GDM will be further explored in the following sections.

**Figure 1:**
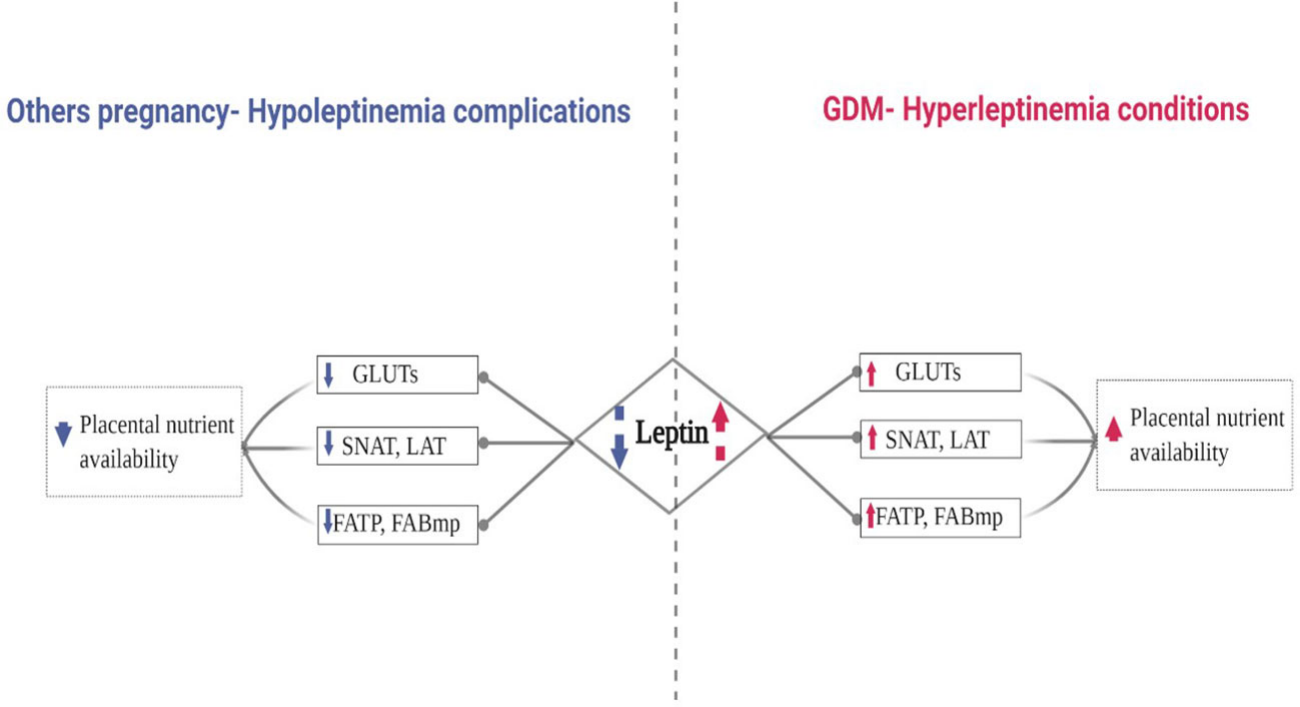
Proposed Algorithm: Leptin’s Role in the Expression of Placental Transporters in Pregnancy Complicated by GDM.

Based on the literature, we hypothesized that the increase of leptin in GDM may enhance placental macronutrient uptake by increasing placental transporter expression. **Figure 1**. represents the proposal algorithm we have adopted in order to support our hypothesis and provide further insight to the role of leptin in GDM and its impact on placental nutrient availability compared to other pregnancy complications related to hypoleptinemia, such as fetal growth restriction (65–67) and spontaneous miscarriage in the first pregnancy trimester (69, 70), as mentioned in the previous section (see **Table 1** for more details).

In order to fulfill the demands of the fetus and normal growth, the human placenta promotes an array of macronutrient transporters, summarized in **Figure 1**. Their expression and activity in response to leptin and in GDM will be discussed in more detail, and further insight will be provided in the next sections.

### 3.3Role of leptin in glucose transport in GDM

Glucose is the primary nutrient required for fetal growth and development. Glucose transport across the placenta to the fetus is mediated by a family of facilitated diffusion transporters, named GLUTs, encoded by the solute carrier family 2 and facilitated glucose transporter (SLC2A) family of genes (83). Seven GLUT isoforms have been reported to be expressed in the human placenta at different sites of the syncytiotrophoblast membrane (84). Placental GLUT activity/expression have been found at distinct sites in the human placenta trophoblast. Most placental GLUTs are expressed in both polarized syncytiotrophoblast membranes of the placenta; in the maternal-facing microvillous plasma membrane (MVM) and the fetal-facing basal plasma membrane (BM), see **Figure 1** for more detail. Leptin has been shown to increase the expression of GLUT1 in human placental trophoblasts under physiological conditions (21). In GDM, leptin could act to further upregulate the expression of GLUT1 and GLUT3 transporters, contributing to hyperglycemia in the fetus.

#### 3.3.1 GLUT1

Glucose transfer from mother to fetus across the placenta takes place primarily through glucose transporter isoform 1 (GLUT1) because of its abundance in both the MVM and BM membranes of the human placenta syncytiotrophoblast (85). GLUT1 plays a regulatory role in the transplacental transport of glucose (86). GLUT1 is the primary glucose transporter protein isoform mediating glucose transport across the syncytiotrophoblast, the transporting epithelium of the human placenta (86). Relevant data related to this isoform have been provided. Thus, the GLUT1 expression is approximately 3-fold greater in the MVM than BM in the syncytiotrophoblast of normal pregnancy conditions (86). On the other hand, conflicting data have been reported, where the activity or expression of GLUT1 differs from first to third trimester of pregnancy (84, 87).

Interestingly, placental GLUT1 mRNA expression was positively correlated with maternal age and inversely correlated with placental weight (88). Moreover, placental GLUT1 protein expression was positively correlated with the pre-pregnancy maternal BMI and umbilical artery glucose levels, but was not associated with umbilical insulin levels (89, 90).

Moreover, GLUT1 activity in the human placenta is likely to be more dependent on maternal glucose metabolism and placenta function. In fact, this may justify the impact of other placental regulatory factors such as leptin, for instance. In term placenta affected by GDM, Gaither et al. reported an increase in GLUT1 expression leading to an increase in glucose uptake in the syncytial BM and no differences with respect to the MVM (85). Regarding the analysis of GLUT1 expression, conflicting studies have demonstrated no differences between placentas affected by GDM and nondiabetic pregnancies (91). Taking all these data in consideration, we can assume that other GDM- related conditions, in addition to insulin-resistance, might be responsible for the altered GLUT1 expression, and that placental leptin or leptin receptor, which are overexpressed in the MVM of human placentas, are affected by GDM, as we have recently shown (20).

**Figure 2:**
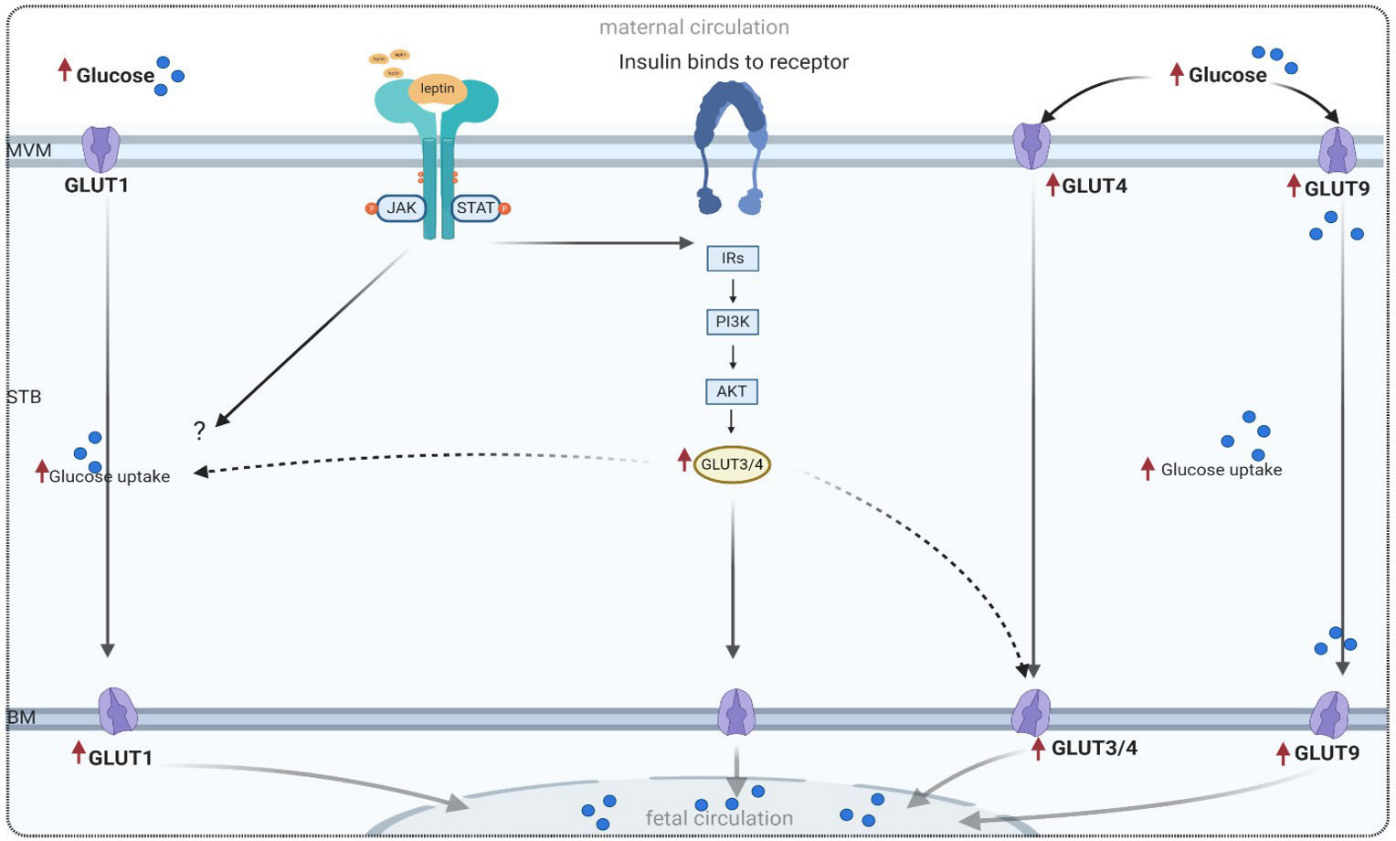
Proposed model of glucose transport in a trophoblast cell, adapted from references listed in **Table 2**. MVM, microvillous plasma membrane; BM, basal plasma membrane; GLUT, glucose transport; PI3K, phosphatidylinositol 3-kinase; IRS, insulin receptor substrate; Akt, protein kinase; mTOR, mammalian target of rapamycin; JAK/STAT, main leptin pathway in trophoblast cell.

GLUT1 expression appears to be inversely related to the maternal glucose concentration; however, within the physiological range, GLUT1 expression is relatively refractory to glucose concentration. Information is still needed on the expression and activity in well-defined conditions of GDM, on the mechanisms and consequences of the changes observed in pregnancy complicated by GDM, and on the role of leptin in regulating placental glucose transport. In this context and regarding GDM, evidence related to the effect of leptin on the expression of GLUT1 is sparse. Thus, the effect of leptin on GLUT1 is indirect. However, as discussed in-depth in recent studies, increased levels of leptin and activation of the leptin receptor in placentas affected by GDM (25–27) may modulate the activity of GLUT1 via the activation of cascade placental signaling pathways such as JAK/STAT or PI3K pathways. Proposal models of this association are shown in **Figure 2**. However, the direct effect of leptin on GLUT1 expression in GDM is unknown, and further investigation is needed to elucidate this effect.

#### 3.3.2 GLUT3

Glucose transporter isoform 3 (GLUT3) provides high-affinity glucose transport to crucial organs and tissues that are highly dependent on a constant supply of glucose, even during hypoglycemia (92). In human trophoblast cells, GLUT3 is present during the first trimester of pregnancy and is characterized by a higher affinity for glucose compared to GLUT1. GLUT3 is an important isoform that ensures an adequate availability of glucose in fetal tissues early in gestation (93). However, there is limited information available on the activity or expression of GLUT3 in human placentas affected by GDM. Only two groups have reported the expression of GLUT3 in human placentas affected by GDM (94, 95). Some investigators have demonstrated a decrease in GLUT3 expression in diabetic groups (mice and human placentas) compared to normal controls (94). In contrast to this data, other studies conducted on animal models (diabetic rats) have shown that placental GLUT3 mRNA and protein levels were increased four-to-fivefold compared to nondiabetic rats (96). These findings suggest that GLUT3 expression may play a major role in placental glucose uptake in GDM by regulating hyperglycemia. Rong et al. discovered decreased GLUT3 gene methylation and increased mRNA expression in GDM patients compared with control pregnant women (97).

Regarding the effect of leptin or LEPR on placental GLUT3 expression, there is a lack of data. However, data from animal models in obese mothers, without diabetic conditions, have demonstrated that leptin and insulin increase GLUT3 expression via activation of the PI3K pathway (98). It is well-established that maternal obesity increases the risk of GDM. These findings support the possible role of leptin in increased glucose transport in GDM.

#### 3.3.3 GLUT4

Another GLUTs family expressed in human placentas is glucose transporter isoform 4 (GLUT4). What is unique about GLUT4 is that it is an insulin-dependent transporter predominantly expressed in adipose tissue, as well as in skeletal and cardiac muscle (99). GLUT4 is also expressed in the MVM of the syncytiotrophoblast of human placentas (100). Xing et al. detected the colocalization of GLUT4 with insulin receptors in term human placental tissue (101). Zang et al. reported a decrease of GLUT4 expression in pregnancy complicated by GDM due to insulin signaling components (IRS-2) and sex hormone-binding globulin (SHBG) impact (102, 103).

On the other hand, the placental GLUT4 protein density was positively correlated with the fetal birth weight in patients with insulin-dependent GDM (89).

Considering the aforementioned data, the activity or expression of GLUT4 might be influenced by other GDM- related conditions in addition to insulin impact. Interestingly, recent research in non-GDM obese pregnancies conducted by Powell and coworkers demonstrated that maternal insulin stimulates placental glucose transport by promoting GLUT4 trafficking to the fetal-facing syncytiotrophoblast BM (104). A possible interpretation of the mechanism might involve the role of leptin and insulin receptor signaling pathways, whose overexpression in GDM have been shown in human placentas (20).

The role of leptin in GLUT4 expression in human placentas complicated by GDM is unknown. However, other studies on animal models, such as heterozygous C57BL6/J-Lepr(db/+) mice that develop spontaneous GDM, have shown that GLUT4 overexpression markedly improves insulin-signaling in GDM, resulting in increased insulin secretion and improved glycemic control (105). These data may indicate a beneficial role of leptin in future GDM therapy.

#### 3.3.4 GLUT8

Glucose transport isoform 8 (GLUT8) is an insulin-dependent transporter similar to GLUT4. It participates in placental glucose transport. Its activity or expression has been confirmed in the syncytiotrophoblast and endothelium of villous vessels of term placentas (106). Currently, the available data on GLUT8 functions and its relationship with fetal growth complications are based on animal models (106, 107). However, there is no data regarding GLUT8 expression in placentas affected by GDM.

#### 3.3.5 GLUT9

Glucose transport isoform 9 (GLUT9) is the only isoform in the GLUT family of glucose transporters that consists of 2 splice variants: GLUT-9a and GLUT-9b, both of which are responsible for transporting glucose and fructose. Additionally, they participate in urate transfer (91, 108). Both forms of the GLUT9 proteins are expressed in the syncytiotrophoblast of the human placenta. Their expression was significantly increased in diabetic placentas from pregnancies complicated by GDM and pre-gestational diabetes (PGDM) compared to healthy controls. Despite the differences in the expression of both GLUT9 forms (109), in trophoblasts, these findings were confirmed by a significant increase in GLUT9 expression in placentas from patients with insulin-dependent diabetes, GDM, and PGDM (110). These findings suggest that GLUT9 expression may play a role in the long-term consequences of fetal overgrowth associated with GDM. Unfortunately, there is a lack of information regarding the link between leptin and leptin receptor activation and their effect on GLUT9 in GDM.

#### 3.3.6 GLUT10

The glucose transporter isoform 10, also known as GLUT10, is characterized by its high affinity for both D-glucose and D-galactose, but not fructose, unlike GLUT9 (111). Recent studies have indicated that DNA methylation regulates GLUT10 gene expression in the human placenta, which may influence the function of other members of the GLUT family in the placenta during pregnancy and in various disease conditions, as suggested by the authors. Additionally, the GLUT10 gene has been found to be involved in a region of human chromosome 20q12-13.1 associated with type 2 diabetes, indicating a potential role for GLUT10 in glucose metabolism and type 2 diabetes (112). To the best of our knowledge, the expression of GLUT10 in normal placentas or placentas affected by GDM has not yet been investigated. Unfortunately, there is currently no available information regarding the role of leptin in its expression.

#### 3.3.7 GLUT12

Glucose transporter isoform 12 (GLUT12) has been reported to be predominantly expressed in the basal membrane of the syncytiotrophoblast and in extra-villous trophoblast cells of the human placenta during the first trimester of pregnancy (113). However, there is currently no available information regarding changes in placental GLUT12 expression related to any form of diabetes.

Furthermore, the existing data regarding changes in the expression of GLUTs during pregnancy and the impact of GDM appear to have conflicting reports. This discrepancy may make it difficult to understand the molecular mechanisms of GDM and further elucidate the role of GLUTs in diabetes-associated conditions. Hyperleptinemia, hyperinsulinemia, and fetal overgrowth (macrosomia) are the primary hallmarks of GDM. Therefore, gaining further insight into their role in placental glucose metabolism and GLUTs expression is necessary to develop new interventions or therapeutic approaches that can improve pregnancy outcomes or at least reduce the exposure of pregnant women and newborns to the consequences of GDM, both in the short and long term.

### 3.4 Role of leptin in amino acids transport in GDM

Concentrations of most amino acids are higher in fetal plasma than in maternal plasma, indicating active accumulation across the syncytiotrophoblast, which is the transporting and hormone-producing epithelium of the human placenta (114). The supply of amino acids to the fetus is critically dependent on the transport capacity of the placenta. This directional transfer requires the coordinated action of over 20 different amino acid transporter proteins, which are localized to both the maternal-facing and fetal-facing plasma membranes of the placental epithelium. These transporters facilitate the uptake of amino acids from the mother and their delivery to the fetus (115, 116). Placental amino acid transport can be categorized based on substrate specificity and sodium dependence, although only two systems have been extensively studied. System A is sodium-dependent and facilitates the uptake of non-essential neutral amino acids, while System L is sodium-independent and facilitates the uptake of essential amino acids in the placenta (17, 117, 118).

#### 3.4.1 System A

**Figure 3:**
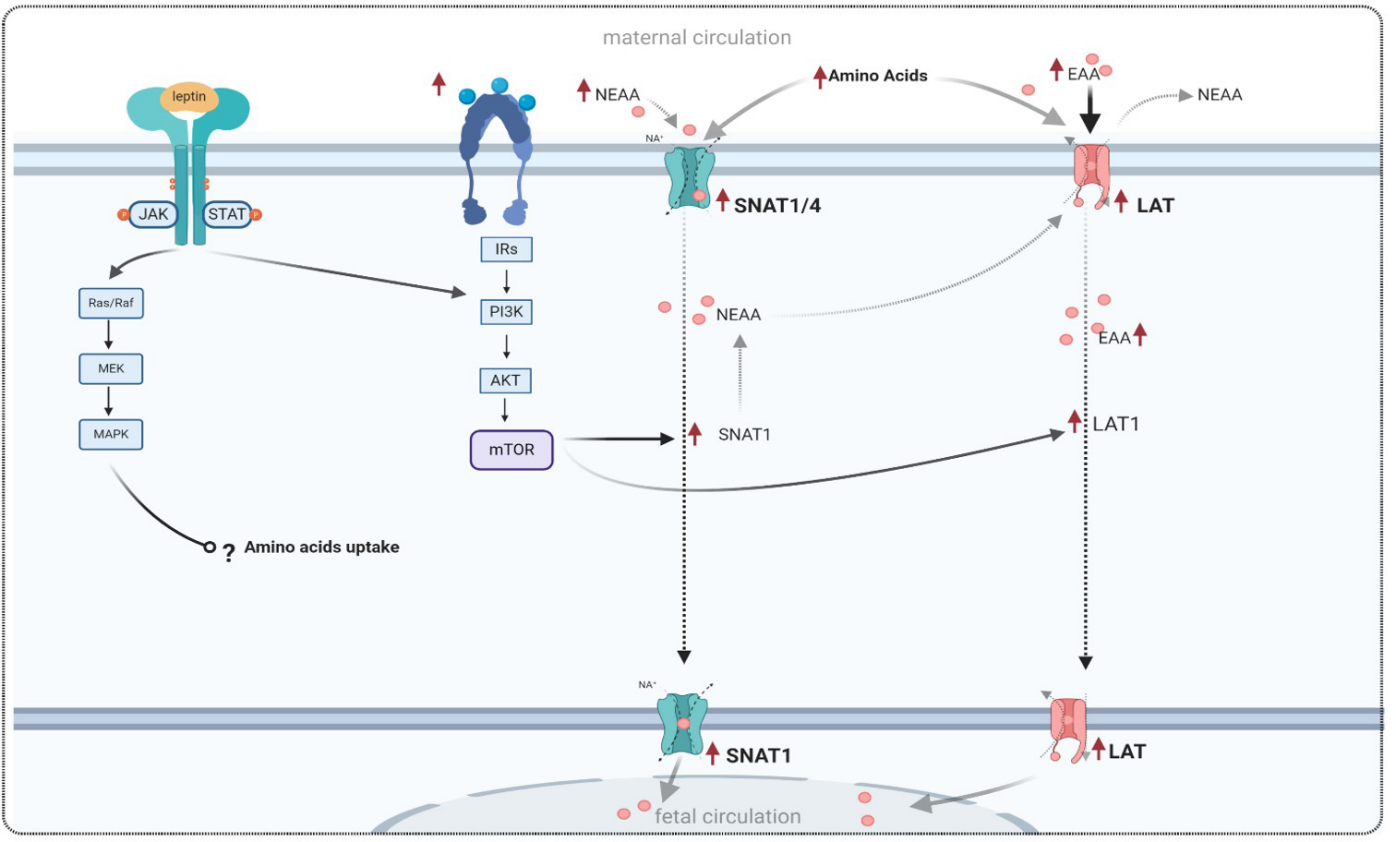
Theoretical model of amino acid transport in GDM conditions, and the effect of leptin signaling on amino acid transporters, adapted from references listed in **Table 2**. Leptin binds to its receptor and activates a cascade of signaling in trophoblast cells, similar to the effects of insulin and the insulin receptor. Leptin and insulin exert their effects by modulating the expression of amino acid transporters via the PI3K and mTOR pathways. NEAA, non-essential neutral amino acid; EAA, essential amino acid; SNAT1, sodium-coupled neutral amino acid transporter 1; LAT1, large neutral amino acid transporter; MAPK, mitogen-activated protein kinases; PI3K, phosphatidylinositol 3-kinase; mTOR, mammalian target of rapamycin.

The System A transporter is responsible for transporting a wide range of non-essential neutral amino acids, including alanine, serine, and glutamine (119). It is highly regulated and consists of three isoforms (SNAT1, SNAT2, and SNAT4), which are encoded by the SLC38A family of genes and expressed in the human placenta (7, 120). System A facilitates the uptake of non-essential neutral amino acids, and SNAT1 is the major contributor to amino acid uptake in cultured primary human trophoblasts. Higher expression of SNAT1 has been positively correlated with birth weight in human placentas from pregnancies affected by GDM, which is associated with fetal macrosomia (121). Regarding the expression of placental amino acid transporters in GDM, some studies have reported a decrease in the expression of system A amino acid transporters in the microvillous membrane (MVM) of placentas from pregnancies complicated by GDM. This decrease may be attributed to a reduction in the number of amino acid transporters, as suggested by the authors (122). However, contrasting these findings, Jansson et al. demonstrated an increase in placental amino acid uptake in human pregnancies complicated by GDM compared to control pregnancies (123). Additionally, the same investigators reported that leptin is involved in the regulation of placental amino acid transport by stimulating the activity of the system A amino acid transporter in term placentas (26) (**Table 2**). In line with this, a recent study has reported that leptin modulates the expression of the system A amino acid transporter in normal placentas through the JAK/STAT pathway (126). A theoretical model of this mechanism is proposed in **Figure 3**.

**Table 2:** Summary of Altered Expressions of Placental Transporters in GDM and Leptin Expressions.

Placental transporters	GDM	Leptin/LEPR	Activated pathways	References
Glucose transporters				
GLUT1	↑	↑ (BeWo cells line)	IRs and PI3K	(85, 95, 119, 124)
GLUT3	↑,↓	NA		(94–96)
GLUT4	↑,↓	↓ (human placenta); ↑ (animal model)	IR and PI3K	(95, 105, 125)
GLUT8	NA	NA	NA	
GLUT9	↑	NA	NA	(91, 109)
GLUT10	NA	NA	NA	
GLUT12	NA	NA	NA	
Amino Acid transporters				
SNAT1	↑	↑	PI3K and mTOR,	(26, 122, 126)
SNAT2	NA	NA	NA	
LAT1-2	↑	↓	PI3K and mTOR,	(119, 127)
Lipids transporters				
FAT/CD36	↑	↑	MAPK	(27, 128)
EL	↑	↑	MAPK & TNF-α	(129)
LPL	↑	NA	NA	(128)
FATP1	↓	NA		(128)
FATP2	↓	↑	NA	(130)
FATP4	↓	NA	NA	(128)
FATP6	↑,↓	↑	NA	(128, 130)
MFSD2A	↓	NA	NA	(131)
FABP1	↑	NA	NA	(132)
FABP4	↑	NA	NA	(128, 133, 134)
FABP5	↑	NA	NA	(133, 134)

↑, increase in expression levels; ↓, decrease in expression levels.

GLUT, glucose transport; SNAT, sodium-coupled or system A for neutral amino acid transporters; LAT, large neutral amino acid transporter; FAT/CD36, fatty acids translocase; EL, endothelial lipase; LPL, lipoprotein lipase; MFSD2A, major superfamily domain 2A; FATP, fatty acid transporter proteins; FABP, plasma membrane fatty acid binding protein; LEPR, leptin receptor; NA, not available or unknown.

#### 3.4.2 System L

**Figure 4:**
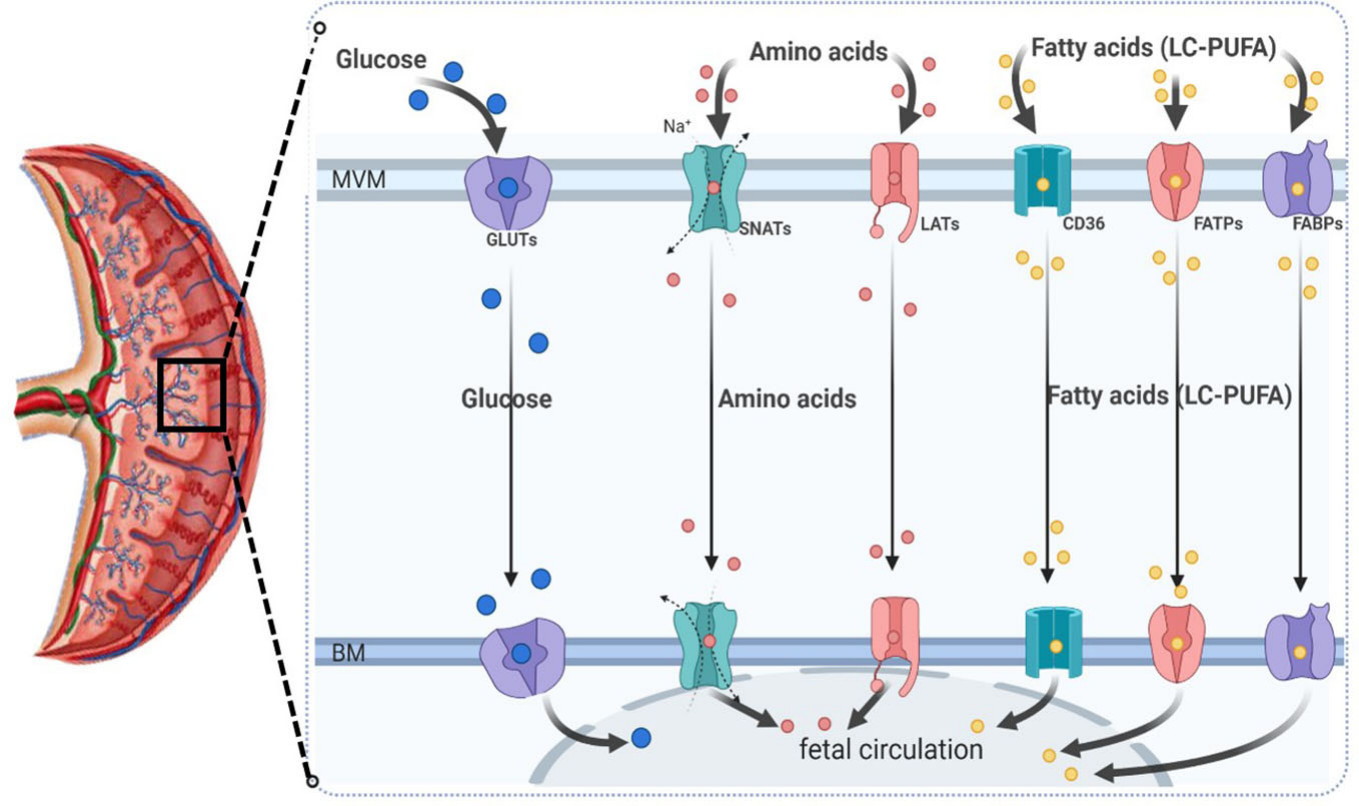
Schematic diagram representing the most relevant placental macronutrient transporters for maternal-to-fetal circulation across the human syncytiotrophoblast. MVM, microvillous plasma membrane; BM, basal plasma membrane; GLUTs, glucose transporters; SNATs, sodium-coupled neutral amino acid transporters; LATs, large neutral amino acid transporter (sodium-independent); CD36, fatty acid translocase; FATP, fatty acid transporter proteins; FABPs, plasma membrane fatty acid binding protein; LC-PUFA, long-chain polyunsaturated fatty acids.

As mentioned previously, the System L or large neutral amino acid transporter system (LAT) is a sodium-independent obligatory exchanger responsible for the transport of neutral amino acids (17, 135). This system primarily transports essential amino acids, such as L-leucine and L-phenylalanine, across the placenta. The System L of amino acid transport consists of two isoforms: LAT1 (also known as SLC7A5) and LAT2. Both isoforms have been found to be expressed in trophoblast cells of the human placenta, and they are localized in the microvillous membrane (MVM) and basal membrane (BM) of the syncytiotrophoblast (7, 118), as shown in **Figure 4**.

Increased activity or expression of both System A and System L in GDM have been reported to accelerate fetal growth and contribute to fetal overgrowth in women with GDM (123, 136), thereby affecting placental function. As mentioned earlier, leptin plays a crucial role in cell proliferation and protein synthesis. In human trophoblastic cells, leptin promotes amino acid synthesis through the activation of MAPK and PI3K pathways (51, 82).

However, changes in placental amino acid transporter expression modulated by leptin, whether through direct or indirect mechanisms, have not been well established, and the available data on this topic are limited.

### 3.5 Role of leptin in lipids transport in GDM

Fatty acids (FAs) play crucial roles in fetal growth and development. An increase in maternal circulating triglycerides (TGs) and dyslipidemia, as well as hyperleptinemia, are associated with GDM complications. These hallmarks of GDM can affect placental FA uptake. The transport of FAs from the maternal circulation to the fetus is mediated by the placenta. Maternal TGs require lipase enzyme activity to cross the placenta as non-esterified fatty acids (NEFAs) and glycerol. Placental NEFAs, under normal conditions, provide the necessary nutrients for fetal development. However, pregnancies complicated by GDM and obesity may be exposed to an excess supply of lipids, leading to an increase in placental FA transporters. This observation has been associated with an increase in fat mass in newborns from mothers with GDM (137).

Maternal FAs can cross the placenta through the microvillous membrane (MVM) of the syncytiotrophoblast via simple diffusion or facilitated transport by FA carriers such as FA translocase (FAT/CD36), FA transport proteins (FATPs), and plasma membrane fatty acid binding protein (FABpm) (7, 135). The expression of these transporters in the placenta facilitates the transfer of NEFAs from the placenta to the fetus through the basal membrane (BM) of the syncytiotrophoblast. However, the available data regarding the expression levels of these transporters in both the MVM and BM of the syncytiotrophoblast are unclear and conflicting in normal physiological pregnancies and altered conditions.

Moreover, placental fatty acid (FA) transport in GDM is more complex and has shown to be more susceptible to alterations in placental factors and pathologic pregnancies related to GDM-induced metabolic changes. In this section, we will focus on the most relevant long-chain polyunsaturated fatty acids (LC-PUFAs) and their related transporters involved in fetal growth and development to gain further insight into the molecular mechanisms altered by GDM pregnancy and the possible role of leptin. As mentioned previously, maternal triglycerides (TGs) need to undergo processing by lipases (7, 18), such as lipoprotein lipase (LPL) and endothelial lipase (EL), in order to cross the placental microvillous membrane (MVM) and basal membrane (BM) and reach the fetus as non-esterified fatty acids (NEFAs). LPL and EL activities are likely required to generate NEFAs from maternal TGs. These two lipases have been found to be highly expressed in the human placenta (138); LPL is particularly abundant in the MVM (132), while EL is present in the membrane of capillary endothelial cells in the human placenta (7, 18, 135).

Fatty acid transport proteins (FATPs) are integral placental transporters for long-chain polyunsaturated fatty acids (LC-PUFAs) such as arachidonic acid (AA) and docosahexaenoic acid (DHA), which are both essential for fetal growth and brain development. The FATP family consists of six isoforms, of which five (FATP1-4, 6) are encoded by the SLC27A gene family (7, 139). All of these isoforms have been reported to be expressed in the human placenta (140). Various studies have indicated that FATP isoforms 1 and 4 are the primary transporters of DHA across the human placenta (18). However, recent research has demonstrated that the sodium-dependent lysophosphatidylcholine symporter (MFSD2A) mediates DHA delivery across the human placenta to the fetus, and its expression may affect placental DHA uptake (131).

In contrast to the FATP family, the placental fatty acid binding protein family (FABPpm) is mainly characterized by its high affinity for LC-PUFAs. It consists of five members (FABP1-5) and is exclusively located in the microvillous membrane (MVM) of the syncytiotrophoblast in the human placenta (140–142). Unfortunately, there is a lack of information regarding FABP expression in the human placenta and in pregnancies complicated by GDM. Nevertheless, some data have been reported regarding altered gene expression of FABP in GDM (133).

The expression and activity of proteins involved in fatty acid transport are influenced by insulin, IGF1, and leptin (69–71). In pregnancies complicated by GDM and a high body mass index (BMI), some alterations in placental fatty acid transport have been observed. These include a decrease in FATP1 and FATP4 expressions, but an increase in FAT/CD36 and FATP6 expressions compared to normal controls (128) (see **Table 1** for more details). These findings suggest that GDM and high BMI increase the uptake of fatty acids in the placenta independent of maternal fatty acid supply, which is reported to be the main factor for fatty acid transport in the placenta. Regarding DHA, a decreased level has been observed in pregnancies complicated by GDM due to low expression of the MFSD2A transporter (131). Interestingly, in contrast to these findings, Ortega et al. have demonstrated unaltered levels of both AA and DHA concentrations in pregnant women with GDM. Additionally, the authors have reported a decrease in most fatty acids in cord serum from GDM subjects, except for α-linolenic acid (ALA), which was higher in the GDM group (143). It is important to note that ALA is one of the most important LC-PUFAs required for fetal growth, as the placenta and fetus cannot synthesize adequate amounts of this molecule to sustain normal fetal development (7).

**Figure 5:**
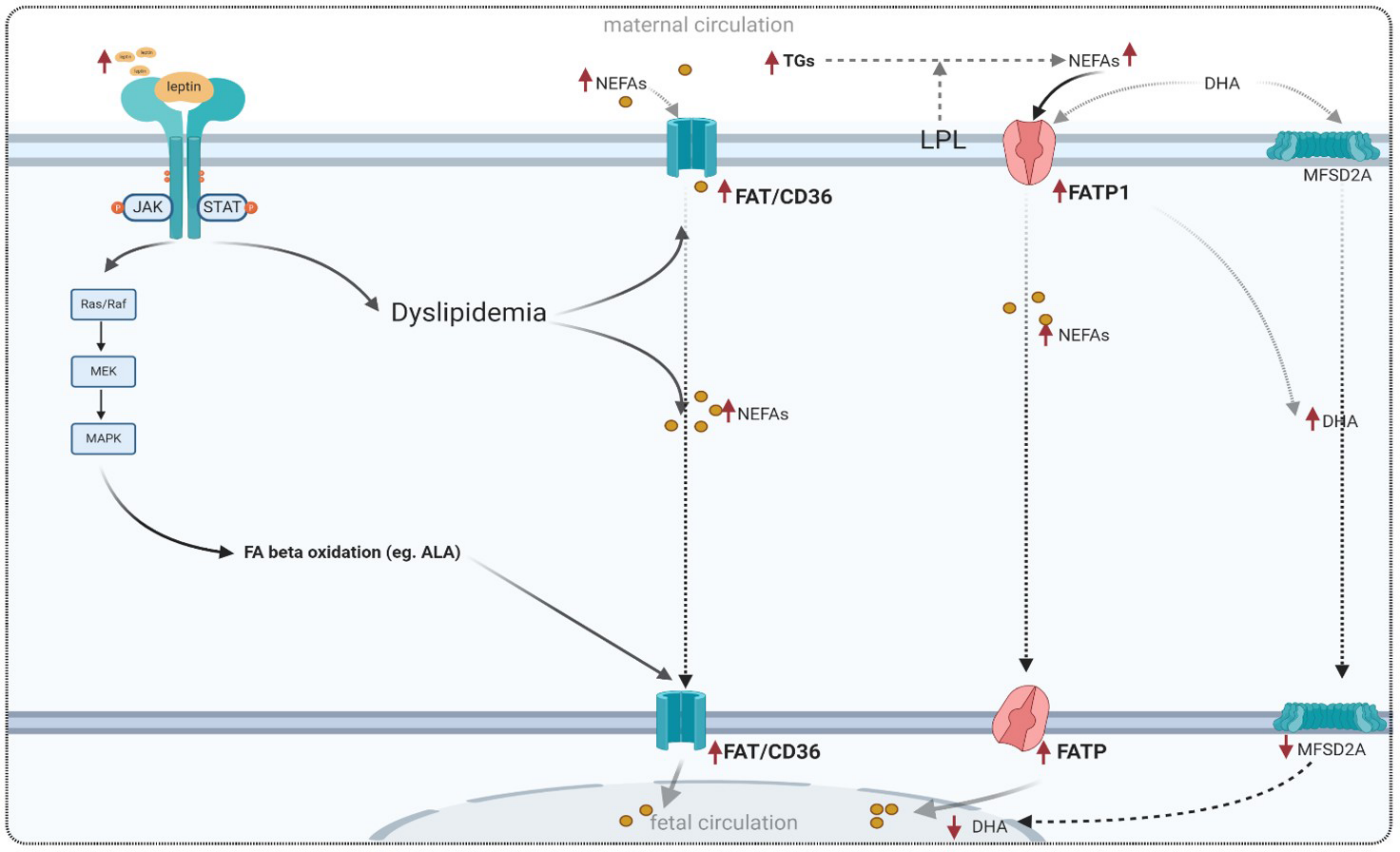
Adapted model for placental fatty acid transport in GDM and the role of leptin signaling in lipid metabolism. FAT/CD36, fatty acid translocase; FATP, fatty acid transporter proteins; FABPs, plasma membrane fatty acid binding protein; MFSD2A, major superfamily domain 2A; DHA, docosahexaenoic acid.

Despite hyperlipidemia associated with GDM and high fetal fat mass, the potential effect of leptin on placental fatty acid transport is not well understood. Leptin and insulin stimulation have been reported to influence fatty acid uptake in BeWo cell placental choriocarcinoma. It has also been shown that an increase in leptin levels leads to an increase in FAT/CD36 expression in cultured samples from the human placenta in obese pregnant women without GDM (27). Leptin may also be related to the antioxidant system. In this context, a recent study has demonstrated that supplementation with 100 mg of alpha-lipoic acid in women with GDM leads to an increase in leptin and adiponectin levels (144). These findings suggest that leptin is likely involved in the regulation of oxidative stress related to GDM. Evidence of this correlation has been mentioned before (76). **Figure 5** summarizes the altered expressions of fatty acid transporters in GDM.

### 3.6 Leptin and GDM: an integrated model of molecular regulatory mechanisms

A wide range of growth factors, pro-inflammatory cytokines, and hormones, including leptin, insulin, and IGF-1, are highly expressed in diabetic placentas in the MVM of the syncytiotrophoblast (10, 82, 122, 137, 145). In pregnancies complicated by GDM, changes in maternal and fetal circulatory levels of leptin are believed to modulate placental functions related to nutrient transport and may potentially alter the expression of placental transporters through intracellular signaling cascades. Therefore, this can be considered the main cause of higher placental nutrient uptake during pregnancy, which can lead to fetal growth disorders such as macrosomia, being large for gestational age (LGA), and an increased risk of metabolic dysfunction in offspring.

**Figure 6:**
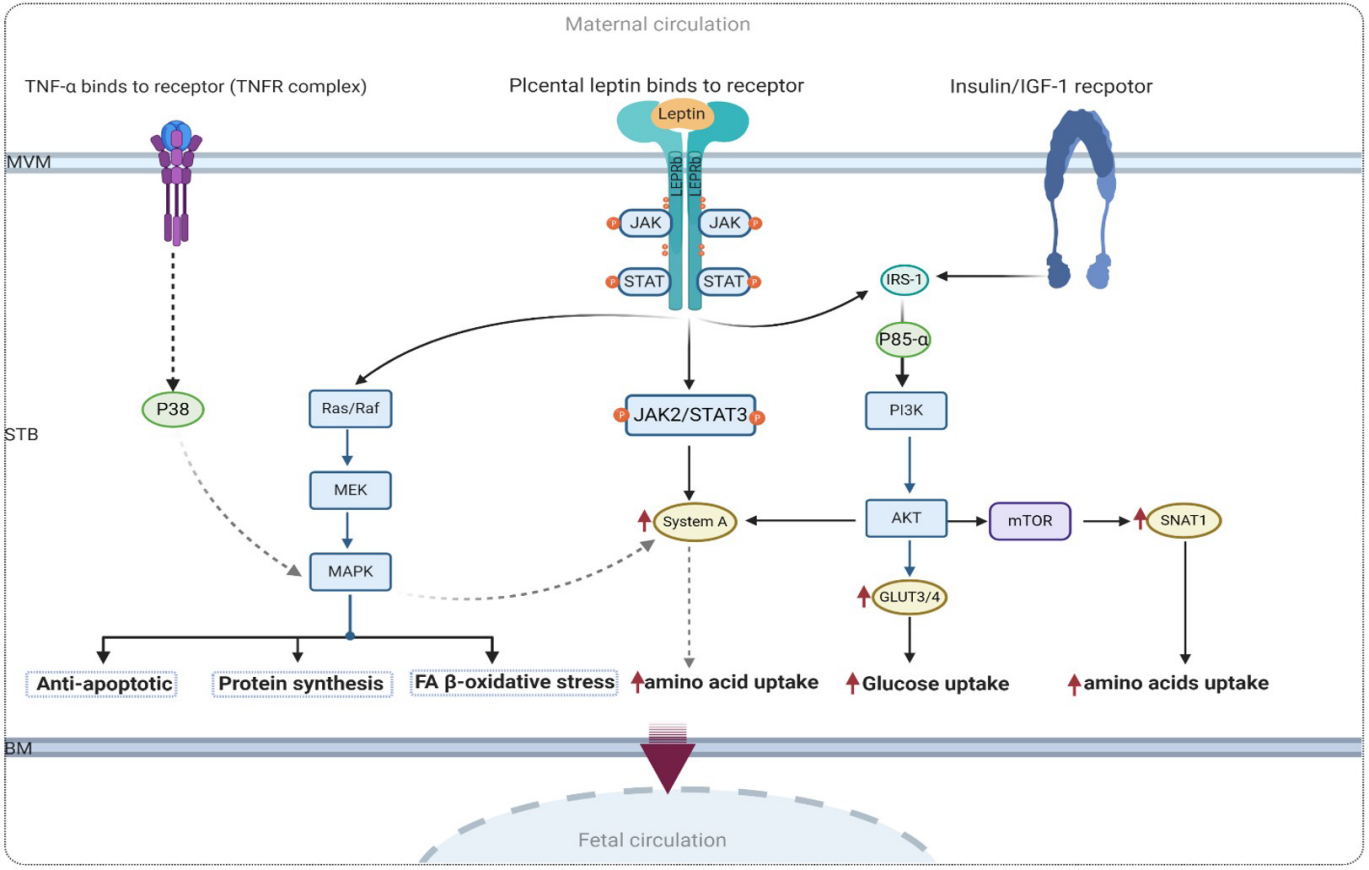
Proposed signaling of leptin and insulin in human trophoblastic cell with GDM. IR-1, Insulin receptor substrate 1; AKT, protein kinase; TNFα, tumor necrosis factor-α; MAPK, mitogen-activated protein kinases; PI3K, phosphatidylinositol 3-kinase; mTOR, mammalian target of rapamycin.

Leptin is a proinflammatory factor that modulates the expression of FA and glycerol transporters and mediates placental NEFA uptake by promoting oxidative stress in GDM conditions, as shown in **Figure 6**. As discussed above, many studies have reported higher leptin levels in GDM pregnancy compared to normal pregnancy. Thus, it is expected that the potential impact of placental leptin signaling on the expression of macronutrient transporters altered in GDM can help elucidate the molecular mechanisms underlying GDM pathophysiology. Furthermore, this may provide insights into the main cause of fetal macrosomia, resulting from changes in the expression pattern of placental transporters.

Leptin serves as both a proinflammatory factor involved in lipid metabolism and GDM pathophysiology, as well as an antiapoptotic factor that acts via the MAPK pathway in the human placenta (50). As mentioned earlier, leptin stimulates system A amino acid transporters through the activation of the JAK2/STAT3 pathway. This provides a compelling reason to investigate other activated signaling pathways through which leptin may modulate glucose uptake and protein synthesis. In this context, the mammalian target of rapamycin (mTOR) is another positive signaling regulator of key placental functions. Given that the mTOR pathway plays a crucial role in nutrient sensing in the human placenta (118), it has been proposed that the activation of placental mTOR signaling may contribute to increased nutrient delivery to the fetus. Therefore, the activation of mTOR and IGF-1 pathways in the placentas of women with GDM who give birth to macrosomic babies has been attributed to the increased expression of SNAT1 (121). A proposed model illustrating the underlying mechanism of mTOR and leptin signaling is presented in **Figure 6**.

Placental leptin binds to the leptin receptor in the MVM of the syncytiotrophoblast and triggers a variety of intracellular functions in the human placenta via the MAPK pathway. Put simply, placental leptin receptor signaling has been shown to promote the MAPK pathway and processes that are important for regulating amino acid metabolism (82) in human trophoblast cells. A recent study demonstrated that an increase in the proinflammatory cytokine TNF-alpha, observed in maternal obesity and GDM, leads to increased amino acid uptake in cultured primary human trophoblast cells in a MAPK-dependent manner. In the context of obesity, it has been shown that leptin stimulates the production of TNF-α in the human placenta (6, 118). Furthermore, Jansson et al. demonstrated that TNF-α regulates placental amino acid uptake by increasing the protein expression of both SNAT1 and SNAT2 isoforms of system A amino acid transporters via p38 MAPK (145). This evidence highlighting the significant association between leptin and TNF-α as proinflammatory mediators in the pathophysiology of GDM may help elucidate the connection between leptin and the MAPK pathway, as well as its role in amino acid and lipid metabolism in diabetic placentas.

**Figure 6** shows the possible molecular mechanism of the link between leptin and proinflammatory TFN-α in the regulation of amino acid metabolism via the MAPK pathway.

## 4 Conclusion

Nutrient transport across the syncytial epithelium of the placenta is a highly regulated process that depends on various nutritional and hormonal signals. In general, obesity and GDM indicate abundant maternal fuel reserves, which stimulate the placental transport of glucose, amino acids, and lipids, thereby increasing their availability for fetal growth and optimizing offspring fitness. The primary objective of this review was to provide an overview of the expression of placental transporters that are altered by GDM, while also assessing the effect of leptin on these transporters and their expression. However, it should be noted that GDM, as a pathophysiological condition, may impact the accuracy of data related to the effect of leptin on placental transporters. Increases in leptin levels during pregnancy complicated by GDM may be associated with an increase in placental nutrient uptake. Nevertheless, the specific effect of leptin on the expression of GLUTs in human placentas affected by GDM remains unclear. Leptin signaling pathways may serve as crucial targets for new GDM therapies. However, further *in vivo* and *in vitro* experiments are required to evaluate the impact of leptin on placental nutrient transport under normal and altered conditions.

## Author contributions

PG, IC and AP-P wrote the paper. All the authors participated in the literature search, in the design of the figures and manuscript revision. All authors have read and agreed to the published version of the manuscript.
